# Special Issue “Migraine and Headache in Children and Adolescents”

**DOI:** 10.3390/life12050704

**Published:** 2022-05-08

**Authors:** Jacob Genizi, Vincenzo Guidetti

**Affiliations:** 1 Bnai Zion Medical Center, Pediatric Department, Haifa 31048, Israel; 2 Bruce Rappaport Faulty of Medicine, Technion, Haifa 31048, Israel; 3 Section of Child and Adolescent Neuropsychiatry, Department of Human Neuroscience, “Sapienza” University of Rome, Piazzale Aldo Moro 5, 00185 Rome, Italy; vincenzo.guidetti@uniroma1.it

Migraine in developmental age is a common pathology. It is estimated that more than 10% of children at the age of 10 suffer from it around the world. In recent years, this disorder has attracted attention for its peculiarities, which often differentiate it from that which occurs in adulthood.

The purpose of this collection is to focus on the different characteristics present in the child and its most common and rarest forms.

Vincenzo Raieli et al. [1] discuss the atypical clinical presentation and possible physio-pathogenetic related aspects of atypical migraine aura. The article analyzes the clinical aspects of pediatric atypical auras, which seem to be more difficult to frame with the mechanisms originally proposed to explain the physio-pathogenetic relationship between cortical spreading depression and aura, and proposes a new terminology: “Multiple, Synchronous and Asynchronous, Cortical and Subcortical Spreading Depression”.

Ilaria Frattale et al. [2], in their narrative review, examined migraine equivalents, which are periodic disorders that can be associated with migraine including; recurrent gastrointestinal disturbance, cyclical vomiting syndrome, abdominal migraine, infantile colic, benign paroxysmal torticollis and benign paroxysmal vertigo.

The gastroenterological episodic syndromes share a common pathogenetic mechanism with migraine due to the same embryologic origin of both enteric and central nervous tissues, which can exert direct effects on each other. In the trigger mechanisms of migraine, a crucial role is played by CGRP (calcitonin gene-related peptide) and PACAP (pituitary adenylate cyclase-activating peptide), that mediate vasodilation, and serotonin, that mediates sensitization of the trigeminal neurons.

Giovanni Prezioso et al. [3] created a consensus document to define a shared clinical pathway between primary care pediatricians and hospitals for the management of children presenting with headache using the RAND/UCLA appropriateness method. Thirty-nine clinical scenarios were developed.

Luigi Francesco Iannone et al. [4] reviewed the emerging pharmacological treatments for migraine in children. They highlighted that the recent introduction of calcitonin gene-related peptide (CGRP) pathway inhibitors and ditans is changing the treatment of migraine; however, the majority of the data are still limited to adulthood. Thus, only a few drugs have indications for migraine treatment in the pediatric age group, and limited evidence gives guidance as to the choice of pharmacotherapy. 

Genizi et al. [5] in a new study on the outcomes of Migraine and Tension-Type Headaches in Children and Adolescents, found that although most pediatric patients presenting with migraine or TTH will experience a favorable outcome over 10 years, only 23–45% will be headache-free. Children with TTH have twice the chance of complete resolution compare to migraine. Regarding diagnosis at follow-up, of the patients with TTH, 36.7% retained their initial diagnosis compared to 59.3% among the migraine patients.

This Special Issue emphasizes the importance and heavy burden that migraine has in the pediatric population. Pediatric migraine has a great impact on school activities, family life and parents’ work. Thus, appropriate resources should be dedicated to educating and treating children and adolescents with migraine.
